# Changes in triglycerides and high-density lipoprotein cholesterol may precede peripheral insulin resistance, with 2-h insulin partially mediating this unidirectional relationship: a prospective cohort study

**DOI:** 10.1186/s12933-016-0469-3

**Published:** 2016-11-04

**Authors:** Tianshu Han, Yu Cheng, Shuang Tian, Li Wang, Xi Liang, Wei Duan, Lixin Na, Changhao Sun

**Affiliations:** National Key Discipline, Department of Nutrition and Food Hygiene, School of Public Health, Harbin Medical University, 157 Baojian Road, Harbin, 150081 People’s Republic of China

**Keywords:** Blood lipids, 2-h insulin, Insulin resistance, Temporal relationship, Mediating effect

## Abstract

**Background:**

Results of longitudinal researches regarding the temporal relationship between dyslipidemia and insulin resistance (IR) are inconsistent. This study assessed temporal relationships of blood lipids with IR and determined whether there are any mediating effects existed in these temporal relationships.

**Methods:**

This study examined a longitudinal cohort of 3325 subjects aged 20–74 years from China with an average of 4.2 years follow-up. Measurements of fasting blood lipids, as well as fasting and 2-h serum glucose and insulin, were obtained at two time points. The Gutt index and HOMA-IR were calculated as indicators of peripheral IR and hepatic IR. A cross-lagged path analysis was performed to examine the temporal relationships between blood lipids and IR. A mediation analysis was used to examine mediating effect.

**Results:**

After adjusting for covariates, the cross-lagged path coefficients from baseline TG and HDL-C to follow-up Gutt index were significantly greater than those from baseline Gutt index to follow-up TG and HDL-C (β_1_ = −0.131 vs β_2_ = −0.047, *P* < 0.001 for TG; β_1_ = 0.134 vs β_2_ = 0.023, *P* < 0.001 for HDL-C). The path coefficients from baseline TG and HDL-C to follow-up 2-h insulin were significantly greater than those from baseline 2-h insulin to follow-up TG and HDL-C (β_1_ = 0.125 vs β_2_ = 0.040, *P* < 0.001 for TG; β_1_ = −0.112 vs β_2_ = −0.026, *P* < 0.001 for HDL-C). 2-h insulin partially mediated the effect of TG/HDL-C on Gutt index with a 59.3% mediating effect for TG and 61.0% for HDL-C.

**Conclusions:**

These findings provide strong evidence that dyslipidemia probably precede peripheral IR and that 2-h insulin partially mediates this unidirectional temporal relationship.

**Electronic supplementary material:**

The online version of this article (doi:10.1186/s12933-016-0469-3) contains supplementary material, which is available to authorized users.

## Background

Both dyslipidemia and insulin resistance (IR) are pathogenetic factors that are fundamental contributors to the development of type 2 diabetes and cardiovascular disease. Although the strong association between these pathogenetic factors has long been recognized [1–3], it is largely unknown which factor is the precursor, or whether the temporal relationship between them is bidirectional [4, 5]. Therefore, it is necessary to clarify the temporal relationship between these factors in order to provide an early and effective target for preventing type 2 diabetes (T2D) and cardiovascular disease (CVD).

The temporal relationship between dyslipidemia and IR is a “chicken-and-egg” question in basic research. Through multiple mechanisms, the metabolites of triglycerides (TG) can interfere with insulin-signaling pathways, while high-density lipoprotein cholesterol (HDL-C) can improve IR in target tissues [6, 7]. On the other hand, IR stimulates lipogenesis and cholesterol synthesis, which results in the overproduction of hepatic very low density Lipoprotein (VLDL), inducing dyslipidemia [8]. Results of longitudinal researches regarding on this issue have also been inconsistent. Some investigators demonstrated that baseline IR was associated with the incidence of dyslipidemia [9, 10]. Alternatively, others demonstrated that blood lipids were a long-term predictor of IR [11]. Notably, in these longitudinal studies, the exclusion of subjects with dyslipidemia or IR traits at baseline based on their study hypothesis favored a one directional result and limited the other directional result (i.e., the exclusion of subjects with dyslipidemia at baseline favors the result that IR precedes dyslipidemia and limits the other result that dyslipidemia precedes IR). This type of study design and statistical analysis are likely the main reasons why the results from these longitudinal studies are inconsistent thus far.

In this study, we adopted a cross-lagged path analysis to explicitly assess the temporal relationship between dyslipidemia and IR using a longitudinal data of China. The cross-lagged path analysis is a model to assess causal associations in data derived from non-experimental, longitudinal research design [12]. This theoretical model has been successfully used to analysis the temporal relationship between inter-related variables in previous studies [13, 14]. According to this theoretical model, if the relationship between dyslipidemia and IR was bidirectional, they would predict each other, and their cross-lagged path coefficients would not be significantly different. However, if IR and dyslipidemia have an underlying causal relationship, the causal variable should predict the consequent variable, and the cross-lagged path coefficient of the causal variable should be significantly greater than that of the consequent variable. We also analyzed whether there were any mediating effects existed in these temporal relationships by mediation analysis in this study.

## Methods

### Study population

The participants were from the Harbin People’s Health Study (HPHS). The HPHS was launched by the Centers for Disease Control and Prevention and the Public Health School in Harbin in 2008 [15]. It covered five urban administrate regions of Harbin. Each region were divided into three strata based on their financial situation and one or two neighborhood committees were chosen from 15 communities that were randomly selected from each stratum in each administrate region by performing a stratified multistage random cluster sampling design. A total of 8940 subjects, aged 20–74 years, were recruited for the study. Subjects were eligible to participate in the study if they had no history of postmenopausal hormone therapy, malignancy, thyroid dysfunction, renal calculi, or corticosteroid or calcitriol use. A total of 4515 subjects (approximately 50.5% of the total subjects) were selected by completely randomized sampling method to participate in a follow-up survey due to the financial limitations of this study. In 2012, 4158 subjects completed the first in-person follow-up survey, for a response rate of 92.1%. The basic information did not differ significantly between the original cohort and the selected cohort. After the exclusion of 398 subjects who were taking hypoglycemic drugs or insulin injections, and 435 subjects who were receiving dyslipidemia treatment at either the baseline or the follow-up survey, 3325 subjects were included in this analysis, with an average follow-up period of 4.2 years. These 3325 subjects included 633 subjects with type 2 diabetes who were not receiving any treatment and 1887 subjects with dyslipidemia who were not receiving dyslipidemia medication at either baseline or the follow-up survey. The study protocols were approved by the Ethics Committee of Harbin Medical University, and written informed consent was provided by all subjects. The methods in this study were in accordance with the approved guidelines.

### Questionnaire survey

Detailed in-person interviews were administered by trained personnel using a structured questionnaire to collect information on demographic characteristics, dietary habits, lifestyles, physical condition and anthropometric characteristics. Current smokers were defined as those who smoked at least 100 cigarettes in a lifetime or smoked every day or currently smoked some days. Current drinkers were defined as those who consumed ≥1 alcoholic drink each month in the 12 months prior to the survey. Regular exercise was defined as any kind of recreational or sport physical activity other than walking for work or life performed at least 30 min for 3 or more days per week.

### Anthropometric measurements

Anthropometric measurements, including height, weight, and waist circumference, were obtained by well-trained examiners, with the participants wearing light, thin clothing and no shoes. Body weight and height were measured to the nearest 0.1 kg and 0.1 cm, respectively. Body mass index (BMI) was calculated as weight (kg) divided by the square of the height in meters (m^2^). An oral glucose tolerance test was performed, according to the World Health Organization guidelines, for each subject.

### Biochemical analyses

Fasting serum lipids, including total cholesterol (TCHO), TG, low-density lipoprotein cholesterol (LDL-C), HDL-C, and fasting and 2-h serum glucose were measured using an automatic biochemistry analyzer (Hitachi 7100, Tokyo, Japan). Fasting and 2-h serum insulin was measured by immunofluorescence method (TOSOH automated enzyme immunoassay (EIA) analyzer AIA-2000ST). Gutt index was calculated as an indicator of peripheral IR, which is based on glucose uptake rates, metabolic clearance rates and mean serum insulin by the following equation: [75,000 + (fasting glucose − 2-h glucose) × 0.19 × body weight]/(120 × log [(fasting insulin + 2-h insulin)/2] × [(fasting glucose + 2-h glucose)/2]) [16]. HOMA models were used to estimate hepatic insulin resistance (HOMA-IR) and beta cell function (HOMA-%β) with HOMA2 calculator updated by the University of Oxford in 2004 [17].

### Outcome measures

Diabetes was identified by self-reports of a history of diabetes diagnosis, fasting blood glucose ≥7.0 mmol/L, and/or 2-h glucose ≥11.1 mmol/L, and/or receiving treatment for diabetes. Dyslipidemia was identified by self-reports of a dyslipidemia diagnosis history, and/or hypercholesterolemia (fasting TCHO ≥6.22 mmol/L, and/or fasting LDL-C ≥4.14 mmol/L), and/or hypertriglyceridemia (fasting serum TG ≥2.26 mmol/L), and/or low HDL-C [fasting serum HDL-C <1.04 mmol/L (male), fasting serum HDL-C <1.29 mmol/L (female)], and/or receiving treatment for dyslipidemia.

### Statistical analysis

All statistical analyses were performed using R 2.15.3 (http://www.r-project.org/) and LISREL 8.52. A two-sided *P* < 0.05 was considered statistically significant. Blood lipids, HOMA-IR, HOMA-%β, Gutt index and insulin were log-transformed to improve the normality of the distribution.

Generalized linear models were performed to test differences in continuous variables between gender and menopause status and calculate covariate-adjusted mean yearly rates of change in Blood lipids, HOMA-IR, HOMA-%β, Gutt index and insulin during the follow-up period. Univariate and multivariate linear regression models were used to determine which type of baseline blood lipids independently predicted future HOMA-IR, HOMA-%β, Gutt index and insulin.

**Fig. 1:**
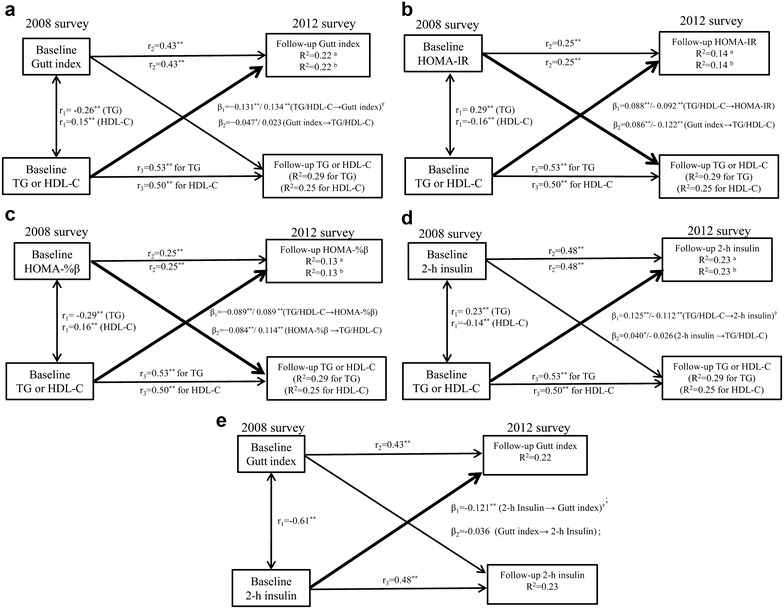
The detailed parameter information on cross-lagged path analysis models. Results were adjusted for age, gender, smoking, alcohol consumption, regular exercise, BMI and caloric intake. **a**–**d** β_1_, cross-lagged path coefficients from baseline TG/HDL-C to follow-up Gutt index; β_2_, cross-lagged path coefficients from baseline Gutt index to follow-up TG/HDL-C; **e**: β_1_, cross-lagged path coefficients from baseline 2-h insulin to follow-up Gutt index; β_2_, cross-lagged path coefficients from baseline Gutt index to follow-up 2-h insulin; r_1_ represents synchronous correlations; r_2_ and r_3_ represents tracking correlations; R^2^: variance explained. ^a^Variance explained when TG included; ^b^ variance explained when HDL-C included; **P < 0.01, *P < 0.05 for coefficients being different from 0; ^†^Difference between β_1_ and β_2_ for being different from 0; TG, triglycerides; HDL-C, high-density lipoprotein cholesterol; BMI, body mass index; Gutt index = [75,000 + (fasting glucose − 2-h glucose) × 0.19 × body weight]/(120 × log [(fasting insulin + 2-h insulin)/2] × [(fasting glucose + 2-h glucose)/2])

Longitudinal changes in blood lipids, HOMA-IR, HOMA-%β, Gutt index and insulin measured at two time points can be modeled using a cross-lagged panel design. In this modeling approach, each variable in the model is regressed on all of the variables that precede it in time. A simplified, conceptual version of the model used in this analysis is presented in Fig. 1. The path coefficient with β_1_ describes the effect of baseline TG or HDL-C on the subsequent Gutt index, and the path coefficient with β_2_ describes the effect of the baseline Gutt index on the subsequent TG or HDL-C. Prior to the cross-lagged path analysis, the baseline and follow-up biochemical indices were adjusted for age, gender, alcohol consumption, smoking, regular exercise, BMI, and caloric intake using a regression residual analysis and then were standardized by Z-transformation (mean = 0, standard deviation, 1). Pearson correlation coefficients of the Z-transformed quantitative variables of biochemical indices at baseline and follow-up were calculated. The cross-lagged path coefficients (β_1_ and β_2_) were estimated simultaneously based on the correlation matrix using the maximum likelihood method in LISREL version 8.52. The percentile confidence interval of cross-lagged path coefficient was estimated using bootstrap simulation for the cross-lagged model. The validity of model fitting was indicated by the root mean square residual (RMR) and comparative fitness index (CFI) [18, 19]. RMR < 0.05 and CFI > 90 indicate relatively good fit to the observed data. The temporal relationships of blood lipids with Gutt index, HOMA-models and 2-h insulin were examined in separate models. The difference between β_1_ and β_2_ derived from the standardized variables was tested using Fisher’s Z-test [14]. Although the significance of individual β_1_ or β_2_ suggests a directional relationship, a significant difference between β_1_ and β_2_ provides stronger evidence for a temporal relationship in the model.

Once the temporal relationships of these biochemical indices had been established, a causal mediation model was constructed to examine whether there are any mediating effects existing in these temporal relationships using R package mediation.

## Results

### The characteristics regarding the study variables

Additional file 1: Table S1 summarizes the mean levels of the study variables at baseline and follow-up by gender and menopausal status. The mean levels of continuous variables with adjustment for age (except age itself) and dichotomous variables were compared between genders and pre- and post-menopausal females.

### The association of baseline blood lipids profile with follow-up IR-related indices by linear regression analysis

Results of univariate and multivariate linear regression models are presented in Table 1. Baseline TG and HDL-C independently predicted the follow-up Gutt index, HOMA-IR, HOMA-%β and 2-h insulin (*P* < 0.05) after adjusting for age, gender, smoking, alcohol consumption, regular exercise, BMI, caloric intake and the baseline dependent variables, whereas TCHO and LDL-C did not. Therefore, based on the result of the linear regression model, we further analyzed the temporal relationships of TG and HDL-C with these indices in the cross-lagged path analysis.

**Table 1:** Linear regression analysis of baseline blood lipids with follow-up IR-related indices

Baseline variable predictors	Follow-up dependent variables	Univariate model^a^			Multiple model^b^		
		β	Std β	*P*	β	Std β	*P*
Ln (TCHO)	Ln (Gutt index)	−0.083	−0.038	0.017	−0.022	−0.010	0.536
	Ln (HOMA-IR)	0.520	0.079	<0.001	0.221	0.031	0.086
	Ln (HOMA-%β)	−0.313	−0.050	0.005	−0.216	−0.030	0.080
	Ln (2-h insulin)	0.079	0.020	0.233	−0.055	−0.014	0.423
Ln (TG)	Ln (Gutt index)	−0.098	−0.131	<0.001	−0.094	−0.125	<0.001
	Ln (HOMA-IR)	0.342	0.154	<0.001	0.294	0.132	<0.001
	Ln (HOMA-%β)	0.345	0.156	<0.001	−0.298	0.135	<0.001
	Ln (2-h insulin)	0.174	0.129	<0.001	0.173	0.129	<0.001
Ln (HDL-C)	Ln (Gutt index)	0.073	0.126	<0.001	0.072	0.124	<0.001
	Ln (HOMA-IR)	−0.215	0.122	<0.001	−0.201	−0.114	<0.001
	Ln (HOMA-%β)	0.212	0.121	<0.001	0.198	0.113	<0.001
	Ln (2-h insulin)	−0.114	−0.109	<0.001	−0.108	−0.104	<0.001
Ln (LDL-C)	Ln (Gutt index)	−0.010	−0.010	0.658	–	–	–
	Ln (HOMA-IR)	0.037	0.013	0.467	–	–	–
	Ln (HOMA-%β)	−0.084	−0.030	0.092	–	–	–
	Ln (2-h insulin)	−0.027	−0.015	0.366	–	–	–

β, regression coefficient; Std β, standard regression coefficient; TCHO, total cholesterol; TG, triglycerides; HDL-C, high-density lipoprotein cholesterol; LDL-C, low-density lipoprotein cholesterol; BMI, body mass index

Gutt index = [75,000 + (fasting glucose − 2-h glucose) × 0.19 × body weight]/(120 × log [(fasting insulin + 2-h insulin)/2] × [(fasting glucose + 2-h glucose)/2])

^a^Each regression model includes a single baseline blood lipid plus age, gender, smoking, alcohol consumption, regular exercise, caloric intake, BMI and dependent variable at baseline

^b^Regression model includes TCHO, TG and HDL-C plus age, gender, smoking, alcohol consumption, regular exercise, caloric intake, BMI and dependent variables at baseline

### The cross-lagged path analysis of TG and HDL-C with IR-related indices

The cross-lagged path coefficients with adjustment for age, gender, smoking, alcohol consumption, regular exercise, BMI and caloric intake are presented in Table 2 and Fig. 1. The path coefficients (β_1_) from the baseline TG to the follow-up Gutt index and 2-h insulin were significantly greater than the path coefficients (β_2_) from the baseline Gutt index and 2-h insulin to the follow-up TG (both *P* < 0.001 for the difference between β_1_ and β_2_). In the TG ↔ HOMA-models analysis, the path coefficients did not differ significantly between β_1_ and β_2_. HDL-C had temporal patterns similar to those noted above for TG. Based on the unidirectional relationships from TG and HDL-C to Gutt index and 2-h insulin, the temporal relationship between Gutt index and 2-h insulin was further examined. The path coefficient (β_1_) from the baseline 2-h insulin to the follow-up Gutt index was significantly greater than the path coefficient (β_2_) from the baseline Gutt index to the follow-up 2-h insulin (*P* < 0.001 for the difference between β_1_ and β_2_).

**Table 2:** The cross-lagged path coefficients with adjustment for covariates

	Path coefficients			Goodness-of-fit model	
	β_1_	β_2_	*P* value^c^	RMR	CFI
Model 1^a^					
TG ↔ Gutt index	−0.131 (−0.168 to −0.099)^**^	−0.047 (−0.072 to −0.010)*	<0.001	0.048	0.916
HDL-C ↔ Gutt index	0.134 (0.103 to 0.167)^**^	0.023 (−0.012 to 0.057)	<0.001	0.021	0.970
TG ↔ HOMA-IR	0.088 (0.055 to 0.121)^**^	0.086 (0.049 to 0.123)**	0.934	0.038	0.934
HDL-C ↔ HOMA-IR	−0.092 (−0.119 to −0.061)**	−0.122 (−0.150 to −0.099)**	0.216	0.040	0.914
TG ↔ HOMA-%β	−0.089 (−0.119 to −0.057)**	−0.084 (−0.109 to −0.060)**	0.837	0.058	0.895
HDL ↔ HOMA-%β	0.089 (0.061 to 0.120)**	0.119 (0.096 to 0.151)**	0.216	0.039	0.941
TG ↔ 2-h Insulin	0.125 (0.090 to 0.159)**	0.040 (0.010 to 0.068)*	<0.001	0.045	0.919
HDL-C ↔ 2-h Insulin	−0.112 (−0.146 to −0.080)**	−0.026 (−0.056 to 0.010)	<0.001	0.041	0.987
Model 2^b^					
2-h insulin ↔ Gutt index	−0.121 (−0.162 to −0.081)**	−0.036 (−0.079 to 0.010)	<0.001	0.059	0.884

Data are cross-lagged path coefficients and its 95% confidence interval with bootstrap simulation

Covariates included age, gender, BMI, alcohol consumption, smoking, regular exercise and caloric intake

TG, triglycerides; HDL-C, high-density lipoprotein cholesterol; BMI, body mass index; RMR, root mean square residual; CFI, comparative fitness index

Gutt index = [75,000 + (fasting glucose − 2-h glucose) × 0.19 × body weight]/(120 × log [(fasting insulin + 2-h insulin)/2] × [(fasting glucose + 2-h glucose)/2])

^a^Model 1: β_1_ describes the path from the baseline TG/HDL-C to the follow-up Gutt index, HOMA-models or 2-h insulin, and β_2_ describes the path from the baseline Gutt index, HOMA-models or 2-h insulin to the follow-up TG/HDL-C

^b^Model 2: β_1_ describes the path from the baseline 2-h insulin to the follow-up Gutt index, and β_2_ describes the path from the baseline Gutt index to the follow-up 2-h insulin

^c^ *P* value for the difference between β_1_ and β_2_

* *P* < 0.05 for β_1_ and β_2_ being different from 0, ** *P* < 0.01 for β_1_ and β_2_ being different from 0

The cross-lagged path analyses were also performed in separate models by gender, menopausal status and HOMA-IR status, with adjustments for age, gender (only for HOMA-IR analysis models), smoking, alcohol consumption, regular exercise, BMI, and caloric intake. The cross-lagged path coefficients did not differ significantly between males and females or between pre- and post-menopausal females (Additional files 1: Table S2 and Table S3). According to the HOMA-IR status, the unidirectional relationship from baseline TG or HDL-C to follow-up Gutt index did not change between the two groups. However, the path coefficients (β_1_) from baseline TG or HDL-C to the Gutt index in the IR group were significantly greater than that in the normal group (Table 3).

**Table 3:** The cross-lagged path coefficients by HOMA-IR status in the total sample, with adjustment for covariates

	Normal (n = 2, 516)		IR (n = 809)^c^		IR status difference	
	β_1_	β_2_	β_1_	β_2_	*P* for β_1_	*P* for β_2_
*Model 1* ^*a*^						
TG ↔ Gutt index	−0.094**	−0.029	−0.173**	0.040	0.047	NS
HDL-C ↔ Gutt index	0.091**	0.016	0.177**	0.016	0.030	NS
TG ↔ HOMA-%β	−0.103*	−0.117*	−0.051	−0.047	NS	NS
HDL ↔ HOMA-%β	0.093**	0.136**	0.114**	0.062	NS	NS
TG ↔ 2-h insulin	0.113**	0.044*	0.128*	0.029	NS	NS
HDL-C ↔ 2-h insulin	−0.094**	−0.020	−0.147**	−0.010	NS	NS
*Model 2* ^*b*^					NS	NS
2-h insulin ↔ Gutt index	−0.095**	−0.023	−0.153**	−0.025	NS	NS

Data are cross-lagged path coefficients

NS, non-significant; TG, triglycerides; HDL-C, high-density lipoprotein cholesterol; BMI, body mass index

Gutt index = [75,000 + (fasting glucose − 2-h glucose) × 0.19 × body weight]/(120 × log [(fasting insulin + 2-h insulin)/2] × [(fasting glucose + 2-h glucose)/2])

Covariates included age, gender, BMI, alcohol consumption, smoking, regular exercise and caloric intake

^a^Model 1: β_1_ describes the path from the baseline TG/HDL-C to the follow-up Gutt index, HOMA-models or 2-h insulin, and β_2_ describes the path from the baseline Gutt index, or 2-h insulin to the follow-up TG/HDL-C

^b^Model 2: β_1_ describes the path from the baseline 2-h insulin to the follow-up Gutt index, and β_2_ describes the path from the baseline Gutt index to the follow-up 2-h insulin

^c^Insulin resistance (IR) was defined as the upper quartile of HOMA-IR

* *P* < 0.05 for β_1_ and β_2_ being different from 0, ** *P* < 0.01 for β_1_ and β_2_ being different from 0

### The mediation analysis

**Fig. 2:**
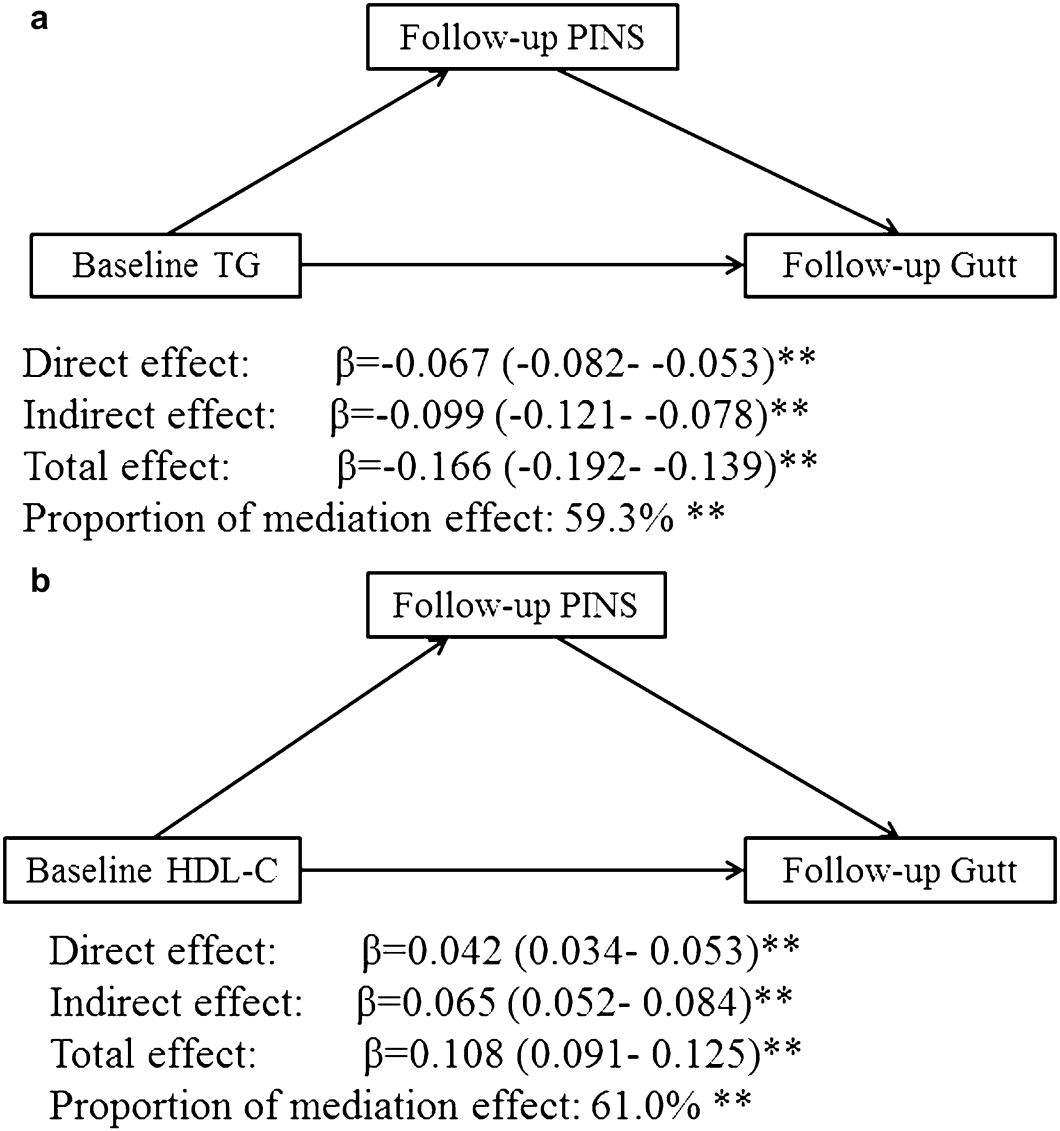
Mediating effects of follow-up 2-h insulin on the association between baseline TG/HDL-C and follow-up Gutt-index. Mediation analysis was used with adjustment for age, gender, BMI, alcohol consumption, smoking, regular exercise and caloric intake. Data are standard regression coefficients and 95% CI. Effect different from 0: **P < 0.01. BMI, body mass index; TG, triglycerides; HDL-C, high-density lipoprotein cholesterol; CI, confidence interval; Gutt index = [75,000 + (fasting glucose − 2-h glucose) × 0.19 × body weight]/(120 × log [(fasting insulin + 2-h insulin)/2] × [(fasting glucose + 2-h glucose)/2])

Figure 2 showed the mediating effects of follow-up 2-h insulin on the unidirectional relationship from baseline TG/HDL-C to the follow-up Gutt index after adjusting for age, gender, smoking, alcohol consumption, regular exercise, BMI and caloric intake. The total effects of TG and HDL-C on the Gutt index were −0.166 for TG and 0.108 for HDL-C (*P* < 0.001). The percentage of the total effects that were mediated by 2-h insulin were estimated to be 59.3% for TG (*P* < 0.001) and 61.0% for HDL-C (*P* < 0.001). Mediation analysis was also performed by HOMA-IR status. The direct effect of TG on Gutt index in the IR group was significantly greater than that in the normal group ($\Delta$ direct effect = 0.0701, 95% CI 0.02–0.114, *P* = 0.02), which reduced the percentage of mediating effect of 2-h insulin.

### The sensitivity analysis

**Fig. 3:**
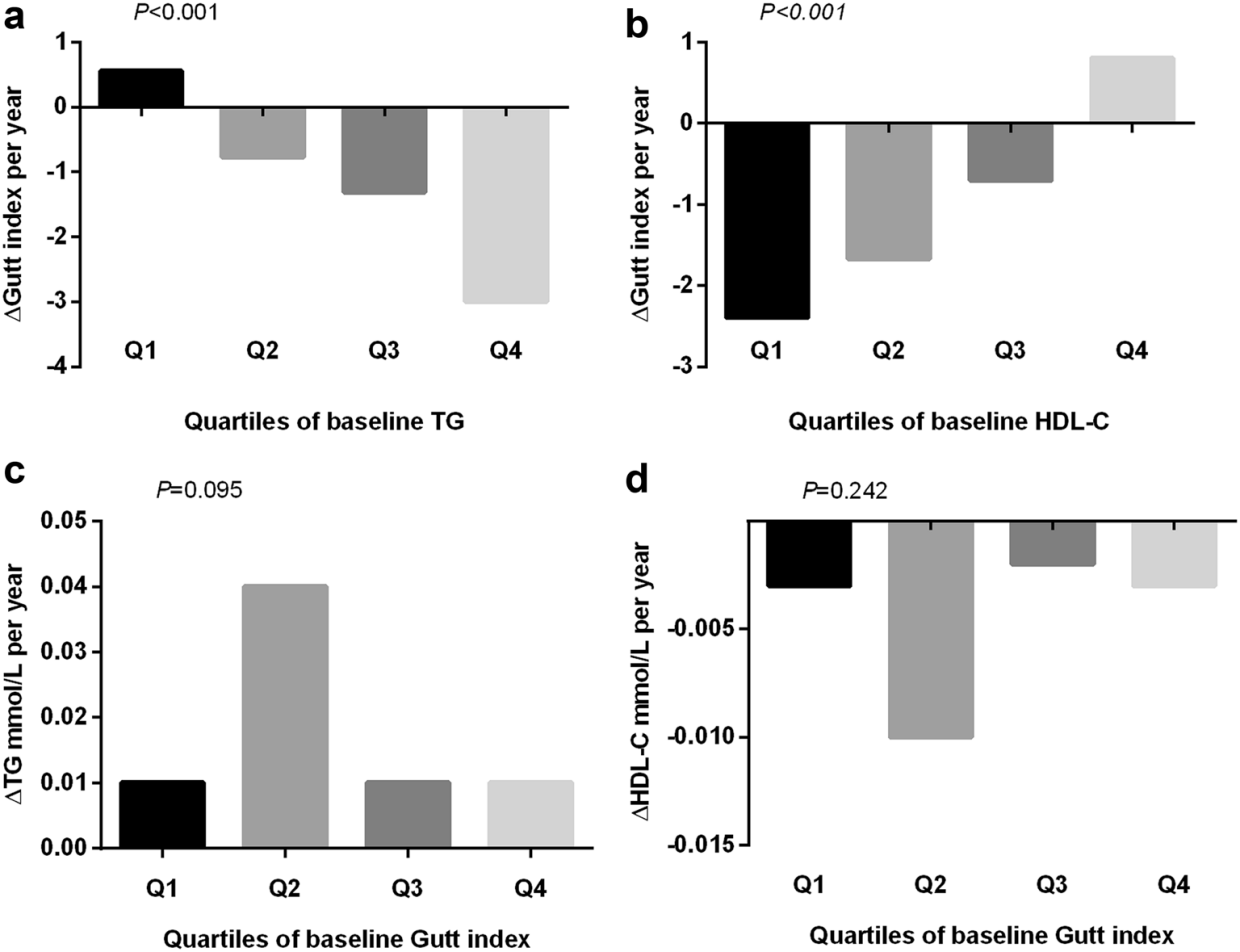
The yearly-rates of change ($\Delta$) in TG, HDL-C and Gutt index by quartiles of their baseline-values. General linear model was used to compare yearly change rates in TG, HDL-C and Gutt index across quartiles of their baseline values, with adjustment for age, gender, smoking, alcohol consumption, regular exercise, BMI and caloric intake. BMI, body mass index; TG, fasting triglycerides; HDL-C, high-density lipoprotein cholesterol; Gutt index = [75,000 + (fasting glucose − 2-h glucose) × 0.19 × body weight]/(120 × log [(fasting insulin + 2-h insulin)/2] × [(fasting glucose + 2-h glucose)/2])

Three sensitivity analyses were conducted in this study. One obtained percentile bootstrap confidence intervals to evaluate sensitivity to the distributions of cross-lagged path coefficients. The second sensitivity analysis examined yearly rates of change (adjusted with age, gender, smoking, alcohol consumption, regular exercise, BMI and caloric intake) in TG, HDL-C, HOMA-models, Gutt index and 2-h insulin according to quartiles of their baseline values using general linear models to validate the results of cross-lagged path analyses (Fig. 3; Additional file 2: Fig. S1, Additional file 3: Fig. S2, Additional file 4: Fig. S3). For example, the yearly rate of change in the Gutt index during the follow-up period significantly varied across increasing quartiles of baseline TG (*P* < 0.001) (Fig. 3a), however, the yearly rate of change in TG did not show a significantly varying trend across quartiles of baseline Gutt index (*P* = 0.095) (Fig. 3c). These results were consistent with the temporal relationships in the cross-lagged models shown in Table 2 and Fig. 1. The third sensitivity analysis used other insulin sensitivity indices such as Stumvoll index and Avignon index instead of Gutt index in the cross-lagged path analysis. The results of the Stumvoll index and Avignon index were similar to that of the Gutt index (Additional file 1: Table S4). Selection of different indices for IR probably did not affect the consequence of these analyses.

## Discussion

In this study, we found for the first time that changes in TG and HDL-C have unidirectional relationships with peripheral IR and bidirectional relationships with hepatic IR by cross-lagged path analysis. 2-h insulin partially mediated the effects of TG and HDL-C on peripheral IR, with a 59.3% mediating effect for TG and a 61.0% mediating effect for HDL-C. Gender and menopausal status had no effect on these temporal relationships.

In this study, we examined the temporal relationships between blood lipids and IR using cross-lagged path analysis, which is a powerful statistical approach in dissecting a causal relationship between inter-correlated variables. Two unidirectional relationships from TG and HDL-C to peripheral IR were confirmed in our analyses, suggesting a potential causal role for TG and HDL-C in the etiology of peripheral IR. Our results are supported by some human studies. For example, IR is not independently associated with future dyslipidemia when visceral adipose tissue included [20], genetic studies have shown that mutations in some genes that only involved in lipoprotein pathways cause dyslipidemia, which consequently lead to IR [21–24], and intervention studies have suggested that controlling dyslipidemia can improve IR [25–27]. Basic and animal studies may provide potential mechanisms of these unidirectional relationships. The metabolites of TG such as free fatty acids (FFAs), diacylglycerol and etc. can regulate insulin-signaling pathways through activating several serine/threonine kinases, which suppress insulin receptor and tyrosine phosphorylation of insulin receptor substrates, inducing peripheral IR [1, 28, 29]. HDL-C can improve peripheral IR probably through multiple mechanisms, including: (1) suppression of inducible nitric oxide synthase and fatty acid synthase; (2) regulation of fat storage in adipocytes via upregulation of the uncoupling protein, and inducing the phosphorylation of AMP kinase in myocytes and liver by its principal apolipoprotein moiety, apoA-1 [30, 31]. In terms of hepatic IR, we found bidirectional relationships of TG and HDL-C with hepatic IR, which were different from that with peripheral IR. Although hepatic IR is frequently associated with peripheral IR, the severity of IR may differ among the various tissues in different individuals [32]. This indicates that treatment for IR should be organ-specific.

Further, we found changes in TG and HDL-C preceded changes in 2-h insulin concentration. The effect of TG/FFA cycle on lipid signaling of β-cells is likely one primary mechanism of these unidirectional relationships [33]. The intermediates within the TG/FFA cycle can increase insulin secretion in β-cells through: (1) activating lipases from islet tissue; (2) activating protein kinase C; (3) alteration of membrane physicochemical properties of β-cells [34–38]. Moreover, FFA forms from hydrolysis of TG can cross β-cell membrane and active β-cell surface fatty acids receptor, which causes an increase in intracellular Ca^2+^, leading to increased insulin secretion [39, 40]. In addition, HDL-C regulates insulin secretion through regulation of cholesterol homeostasis in β-cells via ATP-binding cassette transporters [41, 42]. Increased in HDL-C can reduce β-cell cholesterol content in mice with dyslipidemia, which consequently influence β-cell function [43].

We also found change in 2-h insulin preceded peripheral IR. Catherine Le Stunff et al. showed that early change in postprandial insulin concentration, not in insulin sensitivity, was associated with obesity in early life. This study suggested that postprandial insulin may play an earlier role than peripheral IR in the development of metabolic defects throughout the whole body [44], further supporting our result in this study. Some mechanisms may also support the result that increased insulin concentration preceded peripheral IR. Increased insulin concentration can cause peripheral IR by mediating its own signaling pathway through: (1) diminishing insulin receptors affinity and insulin receptor’s kinase activity, (2) reducing the number of insulin receptors exposed on the cell surface by promoting internalization and degradation of hormone-occupied receptors [45].

Because the unidirectional relationships from TG and HDL-C to 2-h insulin, and from 2-h insulin to peripheral IR were established in this study, these findings prompted us to hypothesize that 2-h insulin concentration probably played mediating roles in the temporal relationships of TG and HDL-C with peripheral IR. The results of mediation analysis in this study did, in fact, show that 2-h insulin partially mediated the temporal relationships with a 59.3% mediating effect for TG and a 61.0% mediating effect for HDL-C. Moreover, hepatic IR probably influences this mediating effect. Hepatic IR causes the overproduction of VLDL, which results in hypertriglyceridemia. Hypertriglyceridemia probably augments the effect of TG on the peripheral IR and reduces the percentage of mediating effect of 2-h insulin.

This study emphasized the important roles of TG and HDL-C as plausible therapeutic targets for improving peripheral IR. It has been suggested that an imbalance in insulin action probably plays a vital role in the development of metabolic abnormalities [46]. The results of this study indicated that changes in TG and HDL-C were likely causal factors of peripheral IR through influencing insulin action. Although TG could represent combined mass of fasting or non-fasting triglyceride-rich lipoproteins [47], increase the risk of CVD and predict ten-year all-cause mortality in patients with T2D [48], the significance of TG as a plausible therapeutic target was underestimated for many years [49]. In China, dyslipidemia has increased significantly during the past decade. The prevalence rates of abnormal lipid levels were estimated at 30.7 and 13.8% for TG and HDL-C [50]. However, the awareness, treatment and control rates of dyslipidemia were estimated at 24.4, 8.8 and 4.3% [51]. It is urgent to initiate dyslipidemia intervention program for improving IR so as to reduce the burden of CVD and T2D.

Although postprandial hyperlipidemia probably played an important role of metabolic defects [52], this study did not include it based on the following reasons. First, the primary objective of this study is to examine the causal relationship between dyslipidemia and IR from epidemiologically point for providing potential effectively treatment target. Fasting lipidemia are more feasible than postprandial lipidemia as treatment targets in the primary care because time and labor intensive are probably two major constraints of postprandial lipidemia measure in large population-based survey. Second, insulin and TG peak frequently occurs at different time points after an oral fat test. It has been reported that TG concentration at 2 h after consumption is not associated with insulin secretion [53]. Third, fasting TG significantly correlates with fasting apolipoprotein B-48 concentration, which may represent the postprandial lipidemia response [54, 55].

## Strengths/weaknesses

This study examined the temporal relationships of blood lipids with IR using a novel theoretical model. Moreover, based on fasting and 2-h status, both hepatic and peripheral IR were included, providing more information for these temporal relationships. Further, this study examined the potential mechanisms of these causal relationships using mediation analysis. However, this study also has some weaknesses. First, this study only included Asian subjects, which is likely to limit the generalizability of our findings to other ethnic populations. Second, hepatic IR and peripheral IR were calculated based on glucose and insulin levels. The results of this study should be confirmed in other studies by more sophisticated methods such as glucose clamp technique and intravenous glucose tolerance test.

In conclusion, by performing a longitudinal assessment of the temporal relationships of blood lipids with IR using cross-lagged path analysis models, this study is the first to find that changes in TG and HDL-C probably precede changes in peripheral IR. A significant causal mediating effect of 2-h insulin on the unidirectional relationships from blood lipids to IR were also demonstrated for the first time. These findings enhance our understanding of the mechanisms of IR. Moreover, these results provide more evidence for the early prevention of IR by improving dyslipidemia.

## Additional files

**Additional file 1: Table S1.** Characteristics regarding the study variables at baseline and follow-up by gender and menopausal groups. **Table S2.** The cross-lagged path coefficients by gender, with adjustment for covariates. **Table S3.** The cross-lagged path coefficients by menopausal status in female sample, with adjustment for covariates. **Table S4.** The cross-lagged path coefficients of TG and HDL-C with other insulin sensitivity indices adjusted for covariates.

**Additional file 2: Fig. S1.** The yearly-rates of change (∆) in TG, HOMA-models and 2-h insulin by quartiles of their baseline-values. General linear model was used to compare yearly change rates in TG, HOMA-models and 2-h insulin across quartiles of their baseline values, with adjustment for age, gender, smoking, alcohol consumption, regular exercise, BMI and caloric intake. BMI, body mass index; TG, fasting triglycerides.

**Additional file 3: Fig. S2.** The yearly-rates of change (∆) in HDL-C, HOMA-models and 2-h insulin by quartiles of their baseline-values. General linear model was used to compare yearly change rates in HDL-C, HOMA-models and 2-h insulin across quartiles of their baseline values, with adjustment for age, gender, smoking, alcohol consumption, regular exercise, BMI and caloric intake. BMI, body mass index; HDL-C, high-density lipoprotein cholesterol.

**Additional file 4: Fig. S3.** The yearly-rates of change (∆) in 2-h insulin and Gutt index by quartiles of their baseline-values. General linear model was used to compare yearly change rates in 2-h insulin and Gutt index across quartiles of their baseline values, with adjustment for age, gender, smoking, alcohol consumption, regular exercise, BMI and caloric intake. Gutt index= [75,000 + (fasting glucose - 2-h glucose) × 0.19 × body weight]/(120 × log [(fasting insulin + 2-h insulin)/2] × [(fasting glucose + 2-h glucose)/2]).

## Abbreviations

BMI: body mass index
CFI: comparative fitness index
FFAs: free fatty acids
HDL-C: high-density lipoprotein cholesterol
HPHS: Harbin People’s Health Study
IR: insulin resistance
LDL-C: low-density lipoprotein cholesterol
RMR: root mean square residual
T-CHO: total cholesterol
TG: triglycerides
VLDL: very low density lipoprotein
