# Supplementary material for: Changes in triglycerides and high-density lipoprotein cholesterol may precede peripheral insulin resistance, with 2-h insulin partially mediating this unidirectional relationship: a prospective cohort study
Source: Cardiovasc Diabetol. 2016 Nov 4;15:154. doi: 10.1186/s12933-016-0469-3 (PMC5095985; doi:10.1186/s12933-016-0469-3)
Supplement: Supplementary file 1 — Additional file 1: Table S1. Characteristics regarding the study variables at baseline and follow-up by gender and menopausal groups. Table S2. The cross-lagged path coefficients by gender, with adjustment for covariates. Table S3. The cross-lagged path coefficients by menopausal status in female sample, with adjustment for covariates. Table S4. The cross-lagged path coefficients of TG and HDL-C with other insulin sensitivity indices adjusted for covariates. [file 12933_2016_469_MOESM1_ESM.docx]

Table S1–Characteristics regarding the study variables at baseline and follow-up by gender and menopausal groups

|  | Gender | | | Menopause | | | Total sample  (3,325) |
| --- | --- | --- | --- | --- | --- | --- | --- |
| Characteristic | Male  (1,055) | Female  (2,270) | *P*-value | Pre-menopause (598) | Post-menopause (1,672) | *P*-value |  |
| Baseline |  |  |  |  |  |  |  |
| Age (years) | 46.6 (11.0) | 46.7 (10.4) | 0.806 | 35.4 (8.4) | 50.8 (7.6) | <0.001 | 46.6 (10.6) |
| BMI (kg/m^2^) | 25.7 (3.3) | 24.6 (3.4) | <0.001 | 23.9 (3.5) | 24.9 (3.3) | 0.145 | 25.0 (3.4) |
| Current smoking [n (%)] | 506 (48.0) | 146 (6.4) | <0.001 | 32 (5.3) | 114 (6.8) | 0.210 | 652 (19.6) |
| Alcohol consumption [n (%)] | 758 (71.8) | 568 (25.0) | <0.001 | 210 (35.1) | 358 (21.4) | <0.001 | 1,326 (39.9) |
| Regular exercise [n (%)] | 573 (54.3) | 981 (43.2) | <0.001 | 278 (46.5) | 703 (42.0) | 0.148 | 1,554 (46.7) |
| Diabetes without medication [n (%)] | 167 (15.8) | 234 (10.3) | <0.001 | 40 (6.7) | 194 (11.6) | 0.001 | 401(12.1) |
| Dyslipidemia without medication [n (%)] | 505 (47.9) | 817 (36.0) | <0.001 | 169 (28.3) | 648 (38.7) | <0.001 | 1,322 (39.8) |
| Caloric intake (kcal/d) | 2,452 (936) | 2,139 (790) | <0.001 | 2,230 (826) | 2,105 (774) | 0.016 | 2,239 (851) |
| Fasting glucose (mmol/L) | 5.11 (1.63) | 4.95 (1.41) | 0.001 | 4.68 (1.34) | 5.04 (1.43) | 0.157 | 5.00 (1.49) |
| Postprandial glucose (mmol/L) | 7.12 (4.32) | 6.71 (3.55) | 0.001 | 5.94 (2.99) | 6.99 (3.69) | 0.031 | 6.84 (3.82) |
| Fasting insulin(mU/L) | 8.34 (5.20) | 8.66 (5.45) | 0.139 | 8.05 (4.89) | 8.89 (5.62) | 0.185 | 8.56 (5.37) |
| Postprandial insulin(mU/L) | 36.1 (28.7) | 39.0 (29.3) | 0.006 | 35.0 (24.2) | 40.5 (30.9) | 0.021 | 38.1 (29.2) |
| TCHO (mmol/L) | 4.93 (0.86) | 4.98 (1.00) | 0.176 | 4.61 (0.97) | 5.12 (0.97) | 0.006 | 4.97 (0.96) |
| TG (mmol/L) | 2.08 (1.52) | 1.65 (1.14) | <0.001 | 1.40 (1.02) | 1.74 (1.17) | 0.414 | 1.79 (1.29) |
| HDL-C (mmol/L) | 1.26 (0.30) | 1.29 (0.31) | 0.017 | 1.30 (0.31) | 1.28 (0.31) | 0.239 | 1.28 (0.31) |
| LDL-C (mmol/L) | 2.75 (0.95) | 2.96 (0.96) | <0.001 | 2.67 (0.96) | 3.06 (0.94) | 0.001 | 2.89 (0.96) |
| Gutt index | 97.1 (48.4) | 96.7 (42.2) | 0.866 | 107.3 (43.7) | 92.9 (40.9) | 0.217 | 96.9 (44.3) |
| Follow-up |  |  |  |  |  |  |  |
| Age (years) | 50.8 (11.0) | 50.9 (10.4) | 0.806 | 39.6 (8.4) | 55.0 (7.6) | <0.001 | 50.8 (10.6) |
| BMI (kg/m^2^) | 26.2 (3.4) | 25.0 (3.5) | <0.001 | 24.0 (4.0) | 25.4 (3.3) | 0.642 | 25.4 (3.5) |
| Current smoking [n (%)] | 422 (40.0) | 124 (5.5) | <0.001 | 26 (4.3) | 98 (5.9) | 0.146 | 546 (16.4) |
| Alcohol consumption [n (%)] | 640 (60.7) | 378 (16.6) | <0.001 | 158 (26.4) | 220 (13.2) | <0.001 | 1,018 (30.6) |
| Regular exercise [n (%)] | 656 (62.2) | 1398 (61.6) | 0.971 | 300 (50.2) | 1098 (65.7) | <0.001 | 2,054 (61.8) |
| Diabetes without medication [n (%)] | 96 (9.1) | 136 (6.0) | <0.001 | 18 (3.0) | 118 (7.1) | 0.014 | 232 (7.0) |
| Dyslipidemia without medication [n (%)] | 211 (20.0) | 354 (15.6) | 0.002 | 75 (12.5) | 279 (16.7) | 0.018 | 565 (17.0) |
| Fasting glucose (mmol/L) | 5.29 (1.71) | 5.05 (1.56) | <0.001 | 4.76 (1.33) | 5.15 (1.62) | 0.574 | 5.12 (1.61) |
| Postprandial glucose (mmol/L) | 6.95 (2.81) | 6.86 (2.76) | 0.236 | 5.99 (2.33) | 7.18 (2.83) | 0.892 | 6.89 (2.77) |
| Fasting insulin(mU/L) | 9.82 (9.00) | 8.89 (6.45) | 0.001 | 8.09 (5.37) | 9.18 (6.78) | 0.929 | 9.19 (7.37) |
| Postprandial insulin(mU/L) | 43.3 (32.8) | 45.5 (32.8) | 0.110 | 38.8 (28.2) | 47.9 (34.0) | 0.011 | 44.8 (32.8) |
| TCHO (mmol/L) | 5.16 (0.91) | 5.39 (1.04) | <0.001 | 5.03 (1.00) | 5.51 (1.03) | <0.001 | 5.31 (1.01) |
| TG (mmol/L) | 2.18 (2.22) | 1.68 (1.09) | <0.001 | 1.49 (1.29) | 1.74 (1.00) | 0.620 | 1.84 (1.56) |
| HDL-C (mmol/L) | 1.15 (0.27) | 1.32 (0.29) | <0.001 | 1.33 (0.30) | 1.31 (0.29) | 0.894 | 1.27 (0.29) |
| LDL-C (mmol/L) | 2.98 (0.79) | 3.13 (0.94) | <0.001 | 2.80 (0.88) | 3.24 (0.93) | <0.001 | 3.08 (0.90) |
| Gutt index | 94.2 (48.2) | 92.2 (42.5) | 0.335 | 106.2 (46.4) | 87.1 (39.8) | 0.003 | 92.8 (44.4) |

Continuous variables are presented as the means (standard derivation).

Generalized linear models and chi-square tests were used to probe for differences in continuous variables (adjusted for age) and dichotomous variables between gender and menopausal groups.

TCHO, total cholesterol; TG, triglycerides; HDL-C, high-density lipoprotein cholesterol; LDL-C, low-density lipoprotein cholesterol; BMI, body mass index; Gutt index= [75,000 + (fasting glucose - 2-h glucose) × 0.19 × body weight]/(120 × log [(fasting insulin + 2-h insulin)/2] × [(fasting glucose + 2-h glucose)/2])

Table S2–The cross-lagged path coefficients by gender, with adjustment for covariates ^a^

|  | Males (n=1,055) | | Females (n=2,270) | | Gender difference | |
| --- | --- | --- | --- | --- | --- | --- |
|  | β_1_ | β_2_ | β_1_ | β_2_ | *P* for β_1_ | *P* for β_2_ |
| Model 1 ^b^ |  |  |  |  |  |  |
| TG ↔ Gutt index | -0.128** | -0.062* | -0.118** | -0.045* | NS | NS |
| HDL-C ↔ Gutt index | 0.152** | 0.030 | 0.125** | 0.044* | NS | NS |
| TG ↔ HOMA-IR | 0.072** | 0.089** | 0.078** | 0.084** | NS | NS |
| HDL-C ↔ HOMA-IR | -0.114** | -0.103** | -0.079** | -0.114** | NS | NS |
| TG ↔ HOMA-%β | -0.089** | -0.072** | -0.082** | -0.088** | NS | NS |
| HDL-C ↔ HOMA-%β | 0.108** | 0.118** | 0.080** | 0.116** | NS | NS |
| TG ↔ 2-h insulin | 0.090* | 0.030 | 0.135** | 0.075** | NS | NS |
| HDL-C ↔ 2-h insulin | -0.135** | -0.032 | -0.101** | -0.048* | NS | NS |
|  |  |  |  |  |  |  |
| Model 2 ^c^ |  |  |  |  |  |  |
| 2-h insulin ↔ Gutt index | -0.111** | -0.047 | -0.125** | -0.026 | NS | NS |
|  |  |  |  |  |  |  |

^a^ Covariates included age, BMI, alcohol consumption, smoking, regular exercise and caloric intake.

^b^ Model 1: β_1_ describes the path from the baseline TG/HDL-C to the follow-up Gutt index, HOMA-models or 2-h insulin, and β_2_ describes the path from the baseline Gutt index, HOMA-models or 2-h insulin to the follow-up TG/HDL-C.

^c^ Model 2: β_1_ describes the path from the baseline 2-h insulin to the follow-up Gutt index, and β_2_ describes the path from the baseline Gutt index to the follow-up 2-h insulin.

* *P*<0.05 for β_1_ and β_2_ being different from 0

** *P*<0.01 for β_1_ and β_2_ being different from 0

NS, non-significant; TG, triglycerides; HDL-C, high-density lipoprotein; BMI, body mass index

Gutt index= [75,000 + (fasting glucose - 2-h glucose) × 0.19 × body weight]/(120 × log [(fasting insulin + 2-h insulin)/2] × [(fasting glucose + 2-h glucose)/2])

Table S3–The cross-lagged path coefficients by menopausal status in female sample, with adjustment for covariates ^a^

|  | Post-menopausal (n=1,672) | | Pre-menopausal (n=598) | | Menopause difference | |
| --- | --- | --- | --- | --- | --- | --- |
|  | β_1_ | β_2_ | β_1_ | β_2_ | *P* for β_1_ | *P* for β_2_ |
| Model 1 ^b^ |  |  |  |  |  |  |
| TG ↔ Gutt index | -0.094** | -0.051* | -0.183** | -0.033 | NS | NS |
| HDL-C ↔ Gutt index | 0.115** | 0.051* | 0.152** | 0.041 | NS | NS |
| TG ↔ HOMA-IR | 0.068** | 0.083** | 0.102* | 0.086* | NS | NS |
| HDL-C ↔ HOMA-IR | -0.066** | -0.095** | -0.118** | -0.165** | NS | NS |
| TG ↔ HOMA-%β | -0.071** | -0.091** | -0.113** | -0.078** | NS | NS |
| HDL-C ↔ HOMA-%β | 0.067** | 0.098** | 0.121** | 0.167** | NS | NS |
| TG ↔ 2-h insulin | 0.124** | 0.069** | 0.161** | 0.096* | NS | NS |
| HDL-C ↔2-h insulin | -0.100** | -0.052* | -0.111** | -0.018 | NS | NS |
|  |  |  |  |  |  |  |
| Model 2 ^c^ |  |  |  |  |  |  |
| 2-h insulin ↔ Gutt index | -0.108** | -0.010 | -0.160** | -0.073 | NS | NS |
|  |  |  |  |  |  |  |

^a^ Covariates included age, BMI, alcohol consumption, smoking, regular exercise and total caloric intake.

^b^ Model 1, β_1_ describes the path from the baseline TG/HDL-C to the follow-up Gutt index, HOMA-models or 2-h insulin, and β_2_ describes the paths from the baseline Gutt index, HOMA-models or 2-h insulin to the follow-up TG/HDL-C.

^c^ Model 2, β_1_ describes the path from the baseline 2-h insulin to the follow-up Gutt index, and β_2_ describes the path from the baseline Gutt index to the follow-up 2-h insulin.

* *P*<0.05 for β_1_ and β_2_ being different from 0

** *P*<0.01 for β_1_ and β_2_ being different from 0

NS, non-significant; TG, triglycerides; HDL-C, high-density lipoprotein; BMI, body mass index

Gutt index= [75,000 + (fasting glucose - 2-h glucose) × 0.19 × body weight]/(120 × log [(fasting insulin + 2-h insulin)/2] × [(fasting glucose + 2-h glucose)/2])

Table S4–The cross-lagged path coefficients of TG and HDL-C with other insulin sensitivity indices adjusted for covariates ^a^

|  | Path coefficients | | | Goodness-of-fit model | |
| --- | --- | --- | --- | --- | --- |
|  | β_1_ | β_2_ | *P*-value ^d^ | RMR | CFI |
| TG ↔ Stumvoll index ^b^ | -0.135** | -0.058** | 0.002 | 0.053 | 0.90 |
| HDL-C ↔ Stumvoll index | 0.130** | 0.047** | 0.001 | 0.042 | 0.91 |
| TG ↔ Avignon index ^c^ | -0.145** | -0.067** | 0.001 | 0.049 | 0.91 |
| HDL-C ↔ Avignon index | 0.133** | 0.062** | 0.003 | 0.047 | 0.93 |
| 2-h insulin ↔ Stumvoll index | -0.192** | -0.010 | <0.001 | 0.057 | 0.88 |
| 2-h insulin↔ Avignon index | -0.212** | -0.094** | <0.001 | 0.054 | 0.90 |

^a^ Covariates included age, gender, BMI, alcohol consumption, smoking, regular exercise and caloric intake.

^b^ Stumvoll index = 0.156 – 0.0000459 × Insulin _120_ – 0.000321 × Insulin _0_ – 0.00541 × Glucose _120_

^c^ Avignon index = [(w × Sib) +Si2h]/2; w=mean Si2h / mean Sib, Sib=10^8^ / (Insulin × Glucose × VD), Si2h=10^8^ / (Insulin × Glucose × VD), where VD=150 ml/kg body weight.

^d^ *P*-value for the difference between β_1_ and β_2_.
