# Supplementary figures and images for: Changes in triglycerides and high-density lipoprotein cholesterol may precede peripheral insulin resistance, with 2-h insulin partially mediating this unidirectional relationship: a prospective cohort study
Source: Cardiovasc Diabetol. 2016 Nov 4;15:154. doi: 10.1186/s12933-016-0469-3 (PMC5095985; doi:10.1186/s12933-016-0469-3)

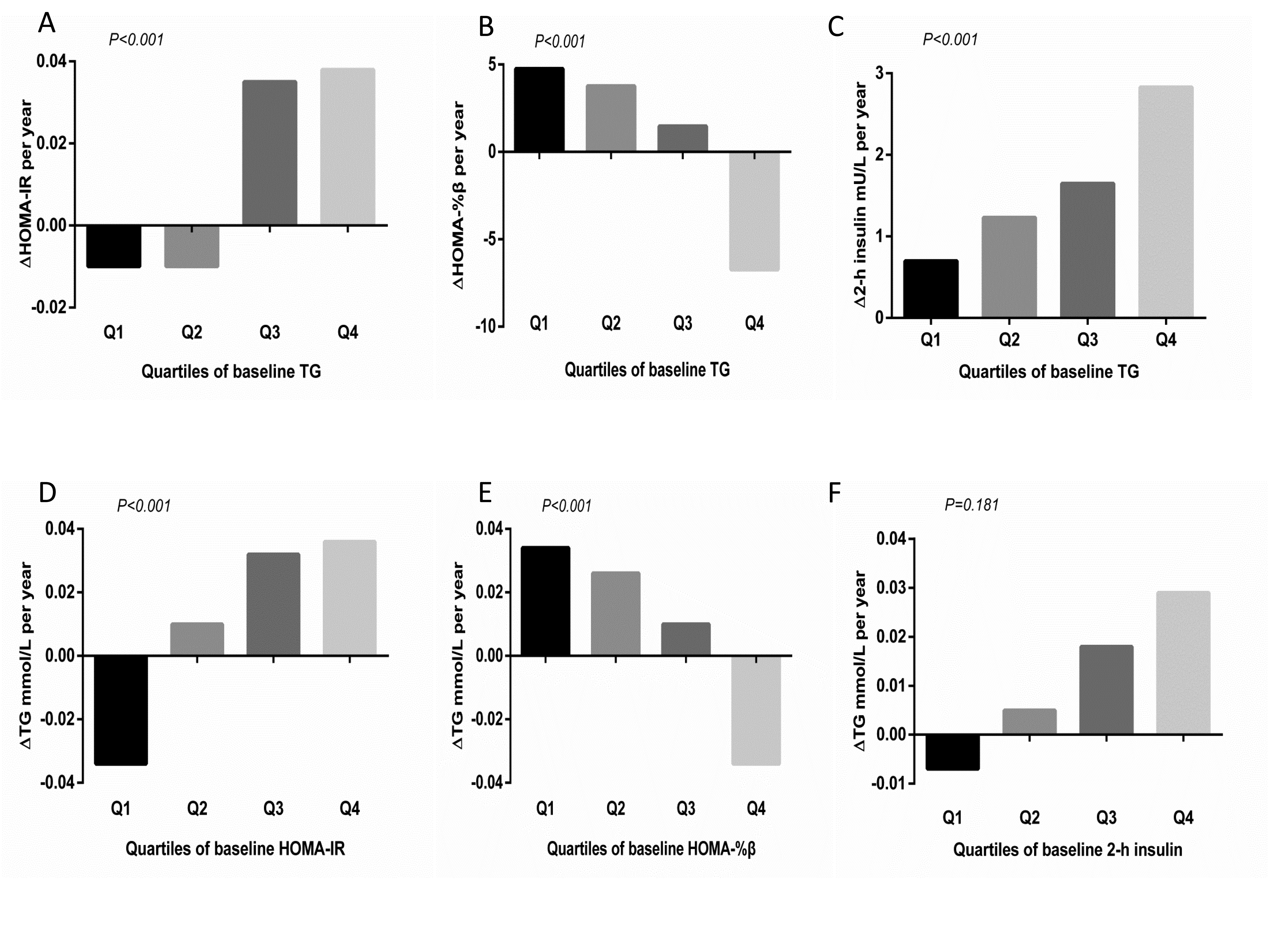

Supplement: Supplementary file 2 — Additional file 2: Fig. S1. The yearly-rates of change (∆) in TG, HOMA-models and 2-h insulin by quartiles of their baseline-values. General linear model was used to compare yearly change rates in TG, HOMA-models and 2-h insulin across quartiles of their baseline values, with adjustment for age, gender, smoking, alcohol consumption, regular exercise, BMI and caloric intake. BMI, body mass index; TG, fasting triglycerides. [file 12933_2016_469_MOESM2_ESM.tif]

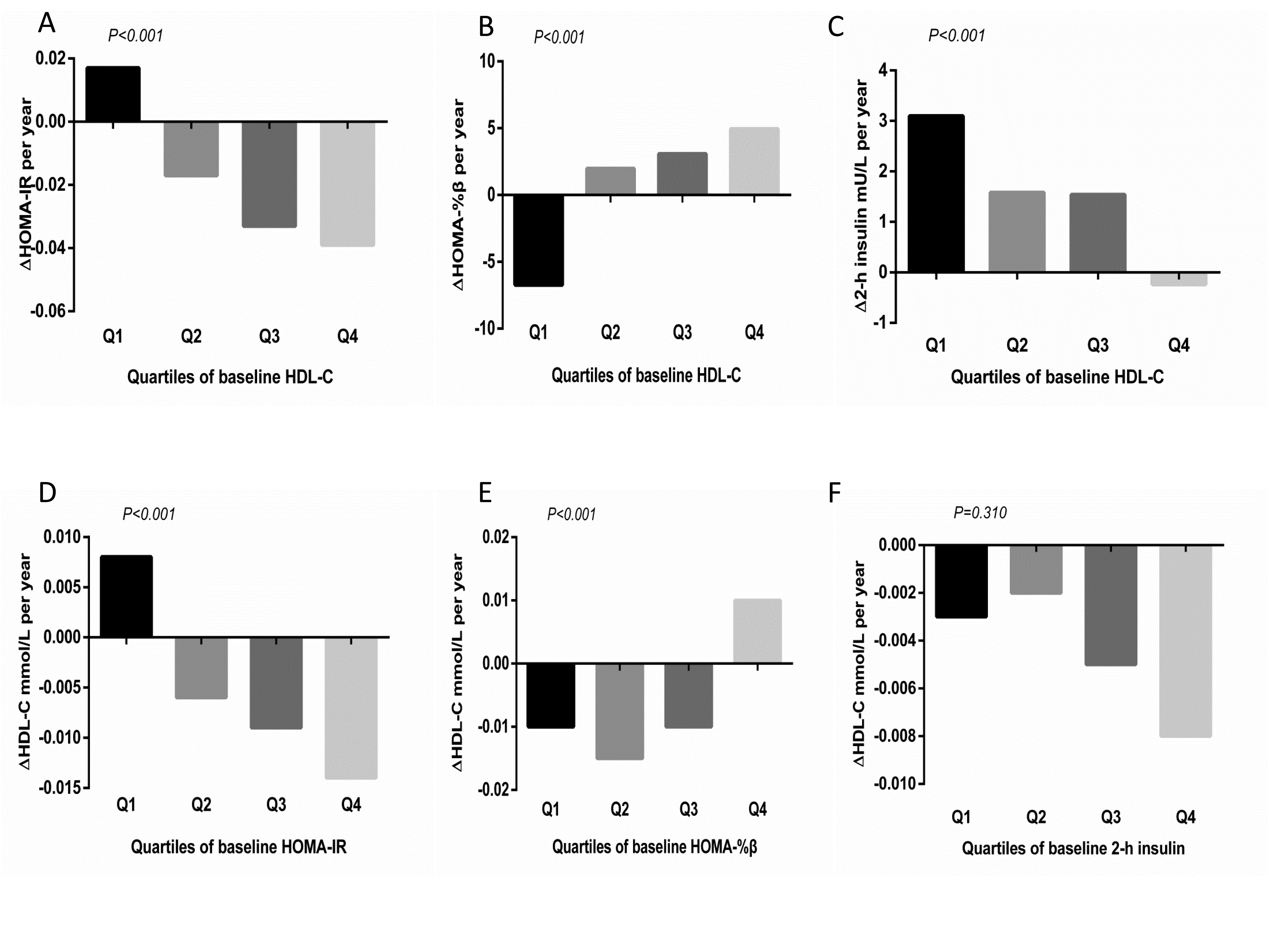

Supplement: Supplementary file 3 — Additional file 3: Fig. S2. The yearly-rates of change (∆) in HDL-C, HOMA-models and 2-h insulin by quartiles of their baseline-values. General linear model was used to compare yearly change rates in HDL-C, HOMA-models and 2-h insulin across quartiles of their baseline values, with adjustment for age, gender, smoking, alcohol consumption, regular exercise, BMI and caloric intake. BMI, body mass index; HDL-C, high-density lipoprotein cholesterol. [file 12933_2016_469_MOESM3_ESM.tif]

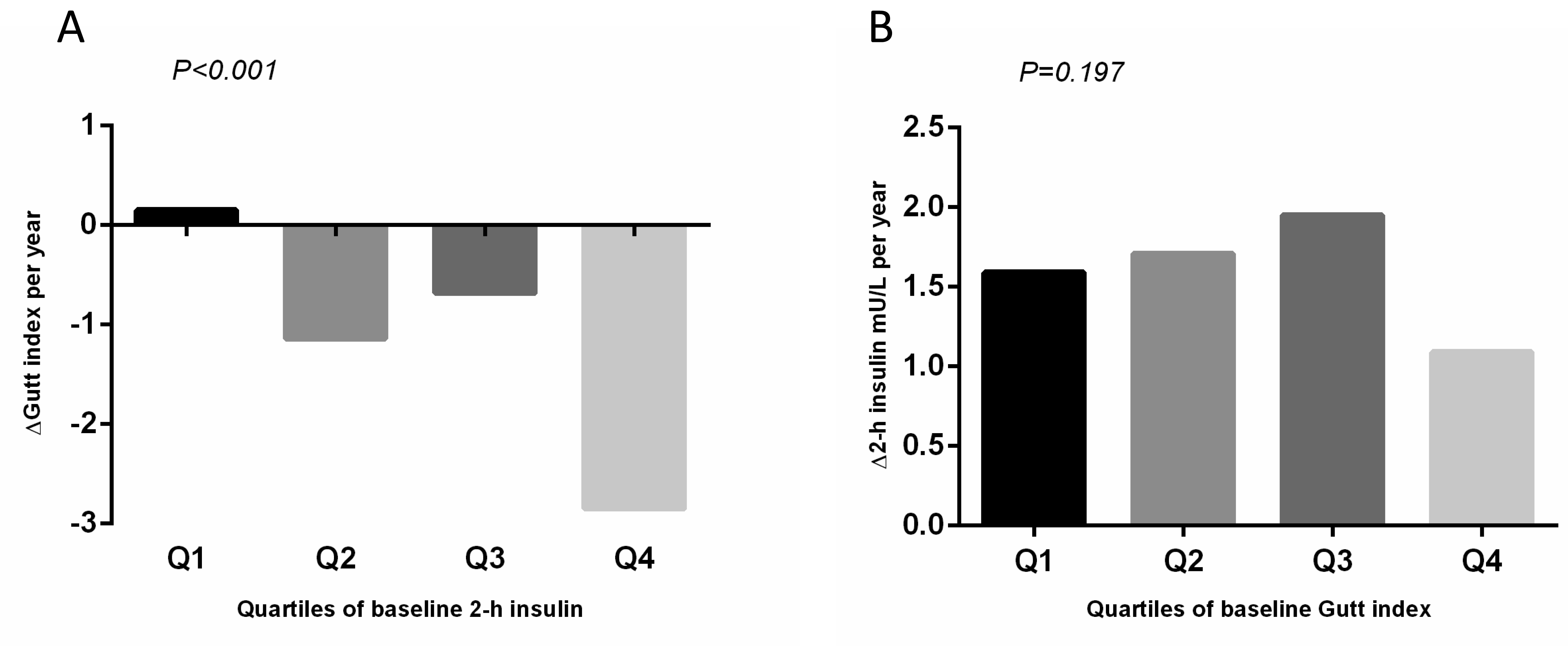

Supplement: Supplementary file 4 — Additional file 4: Fig. S3. The yearly-rates of change (∆) in 2-h insulin and Gutt index by quartiles of their baseline-values. General linear model was used to compare yearly change rates in 2-h insulin and Gutt index across quartiles of their baseline values, with adjustment for age, gender, smoking, alcohol consumption, regular exercise, BMI and caloric intake. Gutt index= [75,000 + (fasting glucose - 2-h glucose) × 0.19 × body weight]/(120 × log [(fasting insulin + 2-h insulin)/2] × [(fasting glucose + 2-h glucose)/2]). [file 12933_2016_469_MOESM4_ESM.tif]
